# Readiness, Acceptability and Use of Remote Monitoring Technologies to Detect Frailty

**DOI:** 10.1093/geroni/igaf122.3441

**Published:** 2025-12-31

**Authors:** Hema Ramamurthi, Greg Kirk, Ann Gruber-Baldini, Denise Orwig

**Affiliations:** Johns Hopkins University, Baltimore, Maryland, United States; Bloomberg School of Public Health, Baltimore, Maryland, United States; University of Maryland School of Medicine, Baltimore, Maryland, United States; University of Maryland, Baltimore, Baltimore, Maryland, United States

## Abstract

Frailty affects a significant portion of older adults and has consistently been shown to lead to adverse health outcomes, especially among People Living with HIV (PLWH). Data from Remote Monitoring Technologies (RMTs) could be used to detect small but significant changes that signal the onset of frailty. Despite the benefits offered by RMTs, there is a paucity of information about essential aspects of RMT use among PLWH. To assess the level of readiness and use of RMTs, we initiated a pilot study to include 30 PLWH aged ≥50 living in the community for a 2-week monitoring period using RMTs (app-based hand grip measurement, motion sensors, accelerometer for gait speed, and digital weighing scale). Surveys included questions about the readiness to use new technology. Of the initial 8 participants, the average age was 61+ 5.3 years, 25% were female, 50% were living alone. Seven participants had access to high-speed internet, and 100% used smartphones. One participant reported using an exercise app, and two reported using a wearable device. The role of technology in providing freedom of mobility was expressed by 50%, and the same respondents somewhat agreed that technology increased productivity. Seven of the 8 participants agreed that people were becoming too dependent on technology and that technological systems are not designed for ordinary people. There was 100% adherence in completing the frailty-related measurements at home. Additional analysis will report on: 1) level of consistency of RMT based measurements to clinic based observations and 2) self-reported acceptability and usability of RMTs.

